# Theaflavins Are Improved by the Oxidation of Catechins in Tannase Treatment During Black Tea Fermentation

**DOI:** 10.3390/molecules30030452

**Published:** 2025-01-21

**Authors:** Lijuan Yang, Mengxue Zhang, Heyuan Jiang, Weiwei Wang, Jigang Huang, Shuixin Ye, Yan Chen, Shuang Liu, Jiaxin Liu

**Affiliations:** 1 Key Laboratory of Special Economic Animal and Plant Biology and Genetic Breeding, Ministry of Agriculture and Rural Affairs, Tea Research Institute, Chinese Academy of Agricultural Sciences, Hangzhou 310008, China; yanglj170699@163.com (L.Y.); zhangmengxue@tricaas.com (M.Z.);; 2 Institute of Urban Agriculture, Chinese Academy of Agricultural Sciences, Chengdu 610299, China; 3 Jiujiang Agricultural Technology Extension Center, Jiujiang 332000, China; 4 College of Food and Health, Zhejiang A & F University, Hangzhou 311300, China

**Keywords:** exogenous tannase, black tea, catechins, theaflavins, oxidation mechanism

## Abstract

The treatment of black tea fermentation with different exogenous tannases was investigated, and processing parameters during black tea fermentation, including fermentation time, fermentation temperature, and exoenzyme amounts, were optimized, while the consumption and transformation pathways of catechins were analyzed. The results showed that tannase from *Aspergillus niger* was ultimately selected as the optimal enzyme to effectively increase the content of theaflavins by promoting the hydrolysis reaction and benzoylation reaction of catechins, resulting in a greater theaflavin (TF) content of 1.41%. The optimal processing conditions were found to be a fermentation time of 3 h, a fermentation temperature of 20 °C, and 1 g of tannase for 300 g of rolled tea leaves. Processing with the exogenous tannase could provide an ideal choice for the efficient utilization of summer and autumn fresh tea leaves, and could be used to develop summer and autumn black tea and to improve the content of theaflavins. It could also be used to develop deep processing of tea products with theaflavin extracts in the future.

## 1. Introduction

Tea originated in southeastern China more than 5000 years ago then gradually expanded to India, Sri Lanka, and many tropical and subtropical countries. Currently, China ranks first in global tea production, followed by India [1]. Tea, as a flavored functional beverage, is commonly consumed by many people daily due to its pleasant sensory attributes [2,3,4], diverse health benefits [5,6,7], and sociocultural traits [8,9]. Tea is currently one of the most consumed beverages in the world and has been very popular for centuries, with consumption rates exceeding those of carbonated beverages, beer, coffee, and wine [10].

Black tea represents over 70% of global tea production, and its consumption holds a dominant position in Western countries [11,12]. However, tea leaves in the summer and autumn are usually rich in polyphenols, and the finished tea often has a stronger bitter and astringent flavor, lacking the sweetness and floral aroma of spring teas. The aforementioned unfavorable factors contribute to the disposal of summer and fall leaves as waste [13]. In the last decade or so, spring green tea production, as well as both summer and autumn black tea production, have been adopted as production peaks in China. Polyphenols are more abundant in summer and fall teas [14], and polyphenols have significant medicinal potential because of their various health benefits. Strategies for processing and using summer tea resources have been reported several times [15].

The key procedure for black tea processing is fermentation, during which polyphenols are oxidized and polymerized to produce compounds such as theaflavins and thearubigins [16,17,18,19]. TFs are very important constituents of black tea. They are the most important substances responsible for the bright and golden rings of black tea soup, and they also influence the freshness of the black tea flavor. The main monomers of TFs include theaflavin (TF), theaflavin-3-gallate (TF-3-G), theaflavin-3′-gallate (TF-3′-G), and theaflavin-3,3′-digallate (TFDG). The available literature suggests that the beneficial health effects and pharmacological activities of TFs mainly include, e.g., skin protection, neuroprotection [20], anticancer [21] and anti-inflammatory effects [22], gut microbiota modulation [23], and antioxidation [24,25], contributing to a wide range of uses in functional foods and pharmaceuticals. However, the content of TFs in black tea samples processed through traditional Chinese processing methods is not high (usually around 0.5%). The formation of TFs during black tea processing is influenced by exogenous factors such as fermentation time [26,27], fermentation temperature [28], and oxygen flux [29]. At the same time, the content and ratio of catechin substrates during tea fermentation are also important endogenous factors for TF formation, such as the ratios of epicatechin (EC), epigallocatechin (EGC), epicatechin gallate (ECG), and epigallocatechin gallate (EGCG), or some combination of them [30,31]. It was found that a 1:2 ratio of EGC to EGCG results in a relatively high formation of TF-3′-G and TF-D-G, which leads to a significant increase in the total content of TFs [32].

Enzymes, as an eco-friendly catalyst, have often been utilized in both primary and further processing to enhance the economic benefits of tea in recent years. Polyphenol oxidases were used to improve the content of TFs, and most TFDG and TF-3′-G were formed; however, they were mostly consumed in the post-production process due to their instability. Certain combinations of polyphenol oxidase and proteinase or pectinase were tested and shown to produce similar results.

Tannase has various applications in the food industry, including its use as a clarifying agent in coffee-flavored refreshing beverages, fruit juices, and wines, as well as in the preparation of water-soluble instant tea [33]. The mechanism is that tannin acyl hydrolase (E.C 3.1.1.20) can hydrolyze the ester bonds of tannins into glucose and gallic acid. It was found that the addition of exogenous tannase not only reduced the bitterness and astringency of green tea beverages [34] but also significantly increased the content of TF (4.7-fold) in black teas [35]. Currently, studies on the oxidation of catechins to form theaflavins during black tea fermentation through exogenous tannase treatments are relatively few. It has been reported that the fermentation time and fermentation temperature have significant interactions with the quality of black tea [36], and they could affect the amount of TF formed in black teas [37]. However, some different enzymes, such as tannase, proteinase, pectinase, and laccase, could catalyze different components to form certain small molecules and could cause certain results beyond expectation through the degradation of specific compounds, e.g., tannins, proteins, pectins, polyphenols, and some others. Therefore, this study investigated the addition of different exogenous enzymes during black tea fermentation and determined the dominant enzymes that could significantly increase the content of TFs in black teas. Concurrently, this study optimized the amount of enzymes added, the fermentation time, and the fermentation temperature for the fermentation of black teas and determined the optimal conditions for the formation of more TFs.

## 2. Results

### 2.1. Effects of Different Exogenous Enzyme Treatments on the Oxidation of Catechins to Form TFs During Black Tea Fermentation

#### 2.1.1. The Variation of Catechins During Black Tea Fermentation

The consumption of four catechins under 10 different enzyme treatments during black tea fermentation is shown in Table 1. The consumptions of all four catechins in different exogenous enzyme treatments during black tea fermentation were different. After 4 h of black tea fermentation, the consumption rates of EGCG, ECG, and EC were relatively high at around 70–90%, but the consumption rate of EGC was only around 30–50%. All treatments by tannase 1, including single or combined enzymes, had increased the consumption of both EGCG and ECG. On the contrary, other enzyme treatments decreased the consumption of EGCG and ECG. The combination treatment by tannase 2, together with pectinase and laccase, could increase the consumption of EC.

#### 2.1.2. Conversion Pathways of Catechins During Black Tea Fermentation

**Figure 1:**
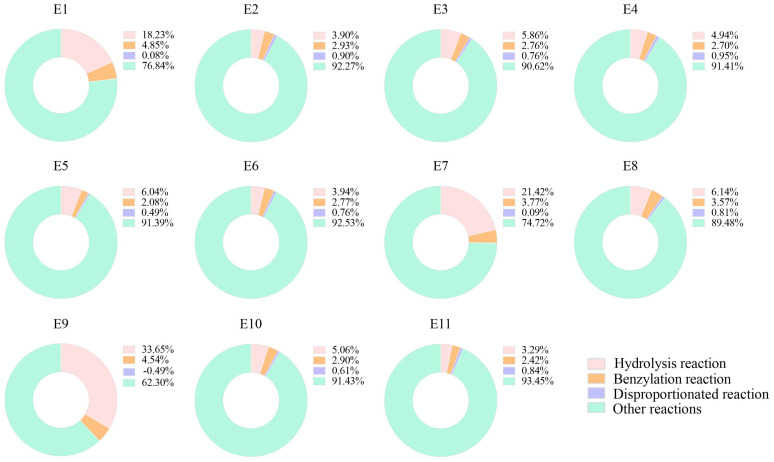
Proportion of Different Reaction Pathways during Black Tea Fermentation. Calculation of the 3 reactions: hydrolysis rate = molar amount of GA/molar amount of 4 catechins, benzoylation rate = molar amount of TFs × 2/molar amount of 4 catechins, disproportionation rate = molar amount of TSA × 2/molar amount of 4 catechins. The 4 catechins are EGCG, ECG, EGC, and EC. E1 to E11 treatments are shown in Table 1. Note: E1 is treatment by tannase 1 (tannase from *Aspergillus niger*); E2 is treatment by tannase 2 (tannase from *Aspergillus oryzae*); E3 is treatment by laccase; E4 is treatment by cellulase; E5 is treatment by pectinase; E6 is treatment by enzyme solution from pear; E7 is treatment by both cellulase and tannase 1; E8 is treatment by both cellulase and laccase; E9 is treatment by both pectinase and tannase 1; E10 is treatment by both pectinase and laccase; E11 is the control.

As can be seen from Figure 1, the hydrolysis reaction pathway of catechins accounted for a larger proportion than both the benzoylation reaction pathway and the disproportionation reaction pathway under all different exogenous enzyme treatments. Compared with the control, the hydrolysis reaction pathway for treatments by tannase 1 and its combination with other enzymes was more dominant and occupied 18% under the treatment by tannase 1 alone and 21% and 33% under the combined treatments by tannase 1 with cellulase or pectinase. It might be related to the fact that pectinase and cellulase were wall-breaking enzymes, which could increase the solubilization of substances. Most different exogenous enzyme treatments promoted the benzoylation reaction, whereas the benzoylation reaction pathway was dominant and increased by 2.5-fold under the treatments by tannase 1 alone or the combination of tannase 1 and pectinase. The disproportionation reaction pathway was slowed down under the treatments by tannase 1 alone or the combination of both tannase 1 and pectinase or cellulase. So, it could be speculated that tannase 1 was the key enzyme to slow down the disproportionation reaction pathway for catechins. Meanwhile, these treatments had the greatest effect on all three reaction pathways, hydrolysis reaction, benzoylation reaction, and disproportionation reaction.

#### 2.1.3. Effect of Different Exogenous Enzymes on the Content of Tea Theaflavins

**Figure 2:**
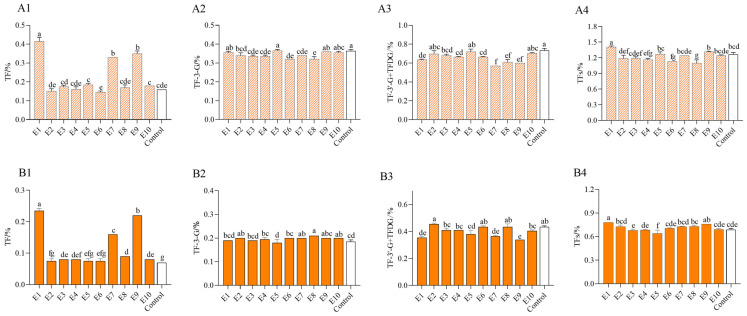
Content of theaflavins treated with different exogenous enzymes during black tea fermentation. (**A1**–**A4**) denote the content of theaflavins at 2 h of black tea fermentation, and (**B1**–**B4**) denote the content of theaflavins at 4 h of black tea fermentation. The data are the mean (±SD) of three replicates, and different letters on the columns denote significant differences (*p* < 0.05). E1 to E10 are shown in Table 1. Note: E1 is treatment by tannase 1 (tannase from *Aspergillus niger*); E2 is treatment by tannase 2 (tannase from *Aspergillus oryzae*); E3 is treatment by laccase; E4 is treatment by cellulase; E5 is treatment by pectinase; E6 is treatment by enzyme solution from pear; E7 is treatment by both cellulase and tannase 1; E8 is treatment by both cellulase and laccase; E9 is treatment by both pectinase and tannase 1; E10 is treatment by both pectinase and laccase.

As can be seen in Figure 2, after 2 h and 4 h of black tea fermentation, treatment by tannase 1 alone significantly increased the content of TF and had the highest content, followed by the combined enzyme treatments by both tannase 1 with pectinase or cellulase which also significantly increased the content of TF. However, the content of TF3G, TF3′G + TFDG was not high during the fermentation under all the exogenous enzyme treatments and was approximately equivalent to that in the control group. After 2 h of fermentation, the black tea fermentation treated by tannase 1 significantly increased the content of TFs and had the highest content of 1.41%. After 4 h of fermentation, the content of TFs during black tea treatment by tannase 1 was still significantly higher than that by other enzyme sources, which indicated that tannase 1 could significantly increase the content of TFs during the fermentation of black tea and was better than other single exogenous enzymes or combined enzymes. The overall content of TFs after 4 h of fermentation was 58% lower than that of tea samples after 2 h. It might indicate that a series of other reactions may be involved after a longer fermentation time, for example, the formation of black tea thearubigins together with the process of theaflavin degradation [38]. It also verified that the fermentation time was an important factor for the accumulation of more theaflavins.

### 2.2. Effect of Tannase on TFs from the Oxidation of Catechins During Black Tea Fermentation

#### 2.2.1. Effect of Temperature and Time on Catechins During Black Tea Fermentation

**Figure 3:**
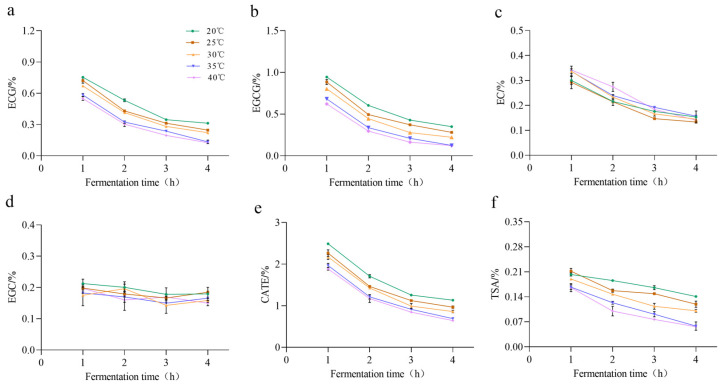
Effect of fermentation time and temperature on the content of catechins. (**a**–**f**) denote the content of ECG, EGCG, EC, EGC, CATE, TSA from 0 to 4 h during black tea fermentation. The data are the mean (±SD) of three replicates.

The results are shown in Figure 3, and it was found that the contents of both EGCG and ECG decreased sequentially with the prolongation of fermentation time. The total amount of catechins also decreased along with the increase in fermentation temperature, as 20 °C > 25 °C > 30 °C > 35 °C > 40 °C, which indicated that catechins were consumed along with the prolongation of fermentation time and the rising of fermentation temperature. However, the variation of both EGC and EC was not obvious. During black tea processing, a variety of dimers were formed by oxidative polymerization, and the main catechin dimer products are theaflavins, theasinensins, and other substances. Theasinensin A (TSA) is the most prominent of theasinensin compounds, which was generated from EGCG by the disproportionation reaction pathway by the action of oxidization enzymes [39]. As shown in Figure 3, the content of TSA exhibited a similar decline with elevated fermentation temperatures and fermentation time, as 20 °C > 25 °C > 30 °C > 35 °C > 40 °C. The rationale for this phenomenon could be attributed to the reduction in EGCG levels resulting from the hydrolysis process by tannase treatment. It could be postulated that higher temperatures would accelerate the hydrolysis process.

#### 2.2.2. Effect of Temperature and Time on Theaflavins During Black Tea Fermentation

**Figure 4:**
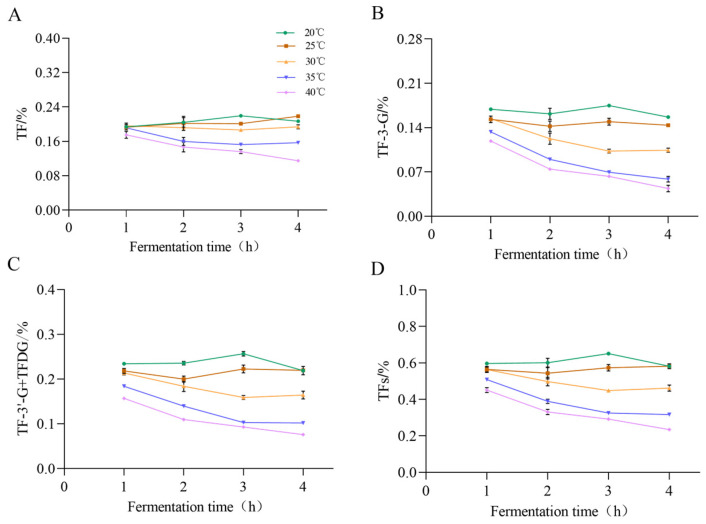
Effect of Fermentation Time and Temperature on the Content of Tea Theaflavins. (**A**–**D**) denote the content of TF, TF-3-G, TF-3′-G + TFDG, TFs from 0 to 4 h during black tea fermentation. The data are the mean (±SD) of three replicates.

As illustrated in Figure 4, the contents of TF, TF3G, TF3′G + TFDG decreased by 20 °C > 25 °C > 30 °C > 35 °C > 40 °C. The higher the fermentation temperature, the lower the theaflavin content. The formation rate of theaflavins was accelerated with the increase in fermentation temperature. However, theaflavins underwent further oxidization and were transformed into other substances during the fermentation, and the content of theaflavins was decreased. The content of TFs showed an increasing trend from 1 h to 3 h under the fermentation temperature of 20 °C and finally decreased after 4 h of fermentation. At the fermentation temperature of 25 °C, they showed an increasing trend from 1 h to 4 h, and the content of TFs was similar with the treatment of the fermentation temperature 20°.

#### 2.2.3. Effect of Tannase Amount on the Content of Catechins and Theaflavins

**Figure 5:**
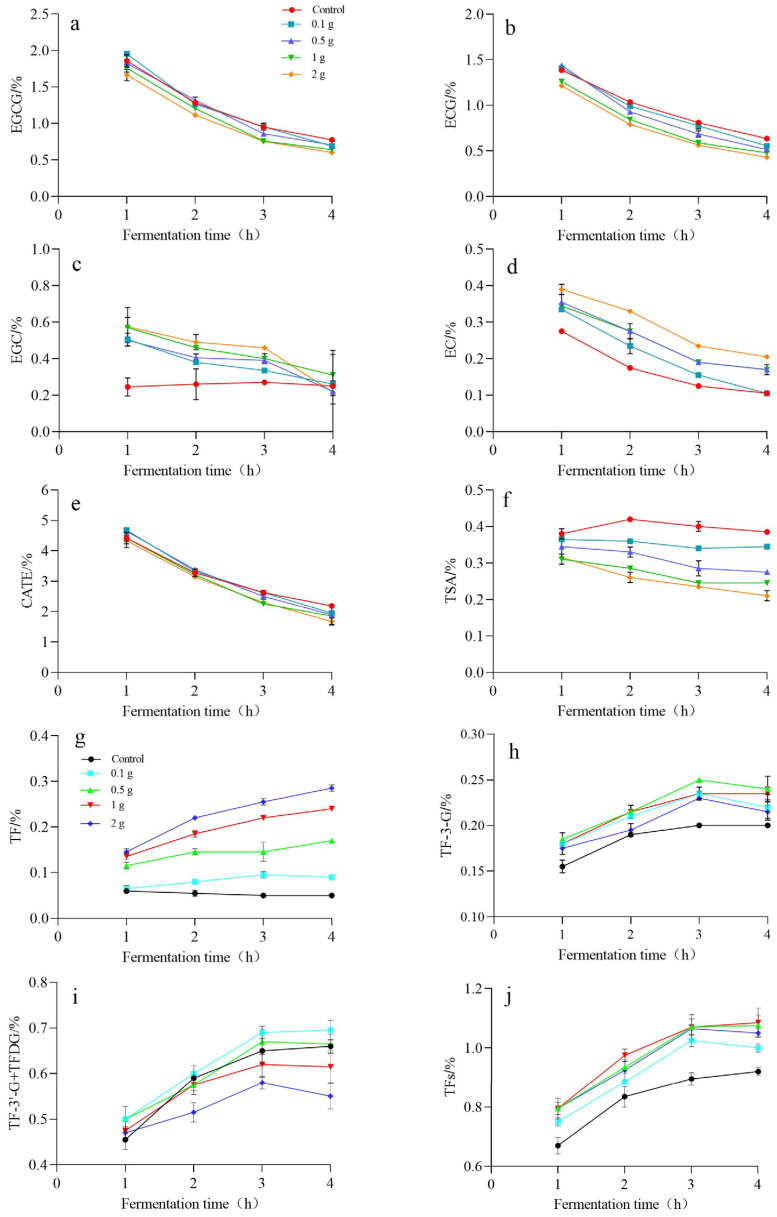
Effect of tannase amounts on catechins and theaflavins. (**a**–**j**) denote the content of EGCG, ECG, EGC, EC, CATE, TSA, TF, TF-3-G, TF-3′-G + TFDG, TFs from 0 to 4 h during black tea fermentation. The data are the mean (±SD) of three replicates.

Experiments were conducted to determine the effect of tannase in different amounts. The results, as can be seen in Figure 5, demonstrated that the addition of tannase was able to increase the content of EC and EGC, as 2 g > 1 g > 0.5 g > 0.1 g > control. On the contrary, the addition of tannase was able to decrease the content of ECG and EGCG. The greater the amount of tannase added, the lower the ECG and EGCG content was in black tea samples. During the fermentation of black tea, EGC was the major flavanol, exhibiting a pronounced increase, because the decrease in EGCG during the fermentation was usually accompanied by an increase in gallic acid and EGC [31]. A greater amount of added tannase could hydrolyze more gallate ester catechins to produce simple catechins, resulting in a decrease in EGCG and ECG, as well as a significant increase in EGC and EC. The addition of tannase was able to significantly reduce the content of TSA, and the ranking of the amount of TSA is control > 0.1 g > 0.5 g > 1 g > 2 g. That might suggest that tannase was able to slow down the disproportionation reaction pathway. Previous studies showed that TF came from the dimerization of EC and EGC, TF3G from EC and EGCG, TF3′G from ECG and EGC, and TFDG from ECG and EGCG [40]. As shown in Figure 5, the addition of tannase significantly increased the content of TF, and the content of EGC and EC was significantly greater than in the control without the addition of tannase. The content of TF3G was also higher than in the control, and the content of TF3′G + TFDG under the 0.1 g tannase addition treatment was always higher than in the control.

The results indicated that, the more tannase that was added, the lower the content of EGCG and ECG. At the same time, EGC preferentially formed TF only. The total content of TFs was higher than in the control, and the content of TFs reached the highest level of 1.12% with the addition of 2 g tannase after 3 h of fermentation, so the addition of tannase could significantly increase the content of TFs. The result was consistent with the previous experimental results.

**Figure 6:**
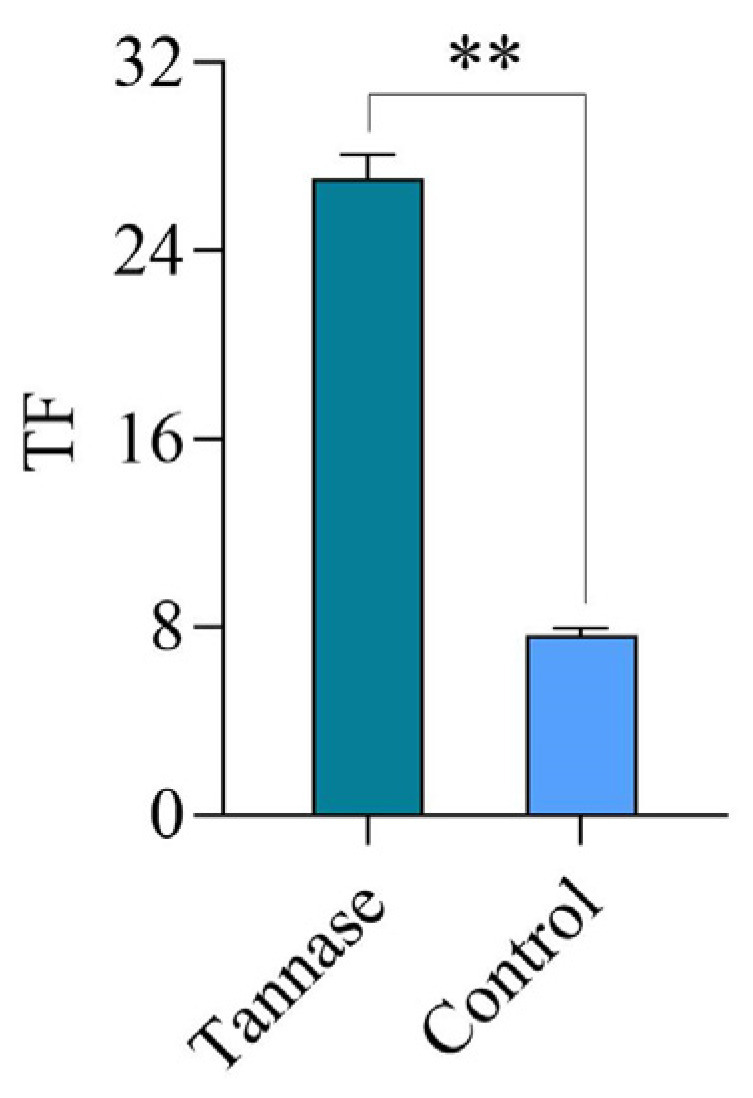
Proportion of TF generated in the benzoylation reaction under the optimal treatment conditions of tannase addition. The data are the mean (±SD) of three replicates, and ** on the columns denote significant differences (*p* < 0.01).

In Figure 6, TF from the benzoylation reaction pathway accounts for 17%, which was twice as much as that of the control, indicating that tannase was able to significantly increase the content of TF.

**Figure 7:**
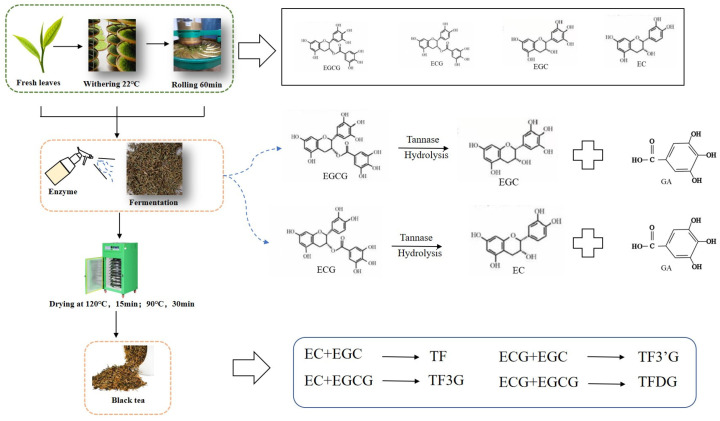
The formation diagram of theaflavins from catechins under tannase treatments during black tea fermentation.

Figure 7 shows the mechanism of TFs increasing from tannase treatment during black tea processing. Usually, both EGCG and ECG are the most two catechin monomers in fresh tea leaves and could be hydrolyzed into EGC and EC by loss of gallic acid groups under the catalysis of tannase. So, more TF could be formed from the combined oxidation of both EGC and EC, because there are more EGC and EC substrates from the hydrolysis of EGCG and ECG. TF is often more stable than other TF compounds, such as TFDG, TF3G, and TF3′G. Finally, the total content of TFs increased during black tea fermentation in this experiment and was significantly higher than in the control.

## 3. Discussion

Black tea production and consumption have been increasing rapidly in China in recent years, and the new processing method by exogenous enzymes has huge potential for application. In China, spring is the busiest season for tea processing. However, the growing seasons of summer and autumn are characterized by higher temperatures and strong sunshine. The contents of polyphenols and caffeine in summer and autumn tea are higher, while the contents of amino acids and aromatic substances are lower, so the quality of summer and autumn black tea produced by the traditional process is poor, often with an astringent taste and insufficient aroma [41]. To improve the quality of summer and autumn black tea, exogenous enzymes might be used for black tea processing [42]. The tannase derived from *Aspergillus niger* has a significant effect on improving black tea theaflavins, with the highest consumption of EGCG, ECG, EC, and EGC during this process. The tannase from *Aspergillus niger* was able to hydrolyze gallate-type catechins to form more non-gallate-type catechins, promote the hydrolysis reaction, and slow down the disproportionation pathway. Treatments by tannase 1 alone, tannase 2 alone, and pectinase combined with tannase 1 could increase the consumption of total catechins. However, tannase 1 treatment alone had the largest consumption of total catechins during black tea fermentation, which was consistent with the previous study showing that tannase hydrolyzed the gallate-ester-type catechins EGCG and ECG, so that EGCG and ECG were predominantly consumed [43]. Liang found that tannase could significantly increase the content of TF, but it should be noted that the production season and cultivar of tea leaves were different from the summer and autumn tea leaves in our experiments [33].

The results showed that the addition of exogenous tannase from *Aspergillus niger* can significantly increase the content of TF monomer, and total TFs were also increased although other theaflavin monomers decreased to some extent. However, it could be developed for the future deep-processed products of tea and will benefit the tea industry.

## 4. Materials and Methods

### 4.1. Materials and Reagents

Fresh tea leaves of the Longjing 43 cultivar and Zhonghuang No. 1 cultivar, with the plucking standard of one bud and two leaves, were harvested in June 2022 at Shengzhou Experimental Base of Tea Research Institute, Chinese Academy of Agricultural Sciences, Shaoxing, Zhejiang Province, China. Fresh tea leaves were collected, sealed, and stored at −18 °C for analysis.

Tannase (300 U/g) was obtained from Cangzhou Xiasheng Enzyme Biotechnology Co. Ltd., Cangzhou, China, as well as acetonitrile (chromatographically pure, Merck, Darmstadt, Germany), sodium carbonate (analytically pure, Yonghua Chemical Technology Co. Ltd., Jilin, China), phosphoric acid, methanol, and foraminol (analytically pure, Shanghai McLean Biochemistry & Technology Co., Ltd., Shanghai, China); catechin (C), epicatechin (EC), gallocatechin (GC), epigallocatechin (EGC), epicatechin gallate (CG), epicatechin gallate (ECG), gallocatechin gallate (GCG), and epigallocatechin gallate (EGCG) (purity for catechin monomers: >98%, Sigma Co., St. Louis, MO, USA); theaflavin (TF1), theaflavin-3-gallate (TF2A), theaflavin-3′-gallate (TF2B), and theaflavin-3,3′-digallate (TF3) (purity for theaflavins monomers > 98%, Wako Pure Pharmaceuticals Co. Industries, Ltd., Shanghai, China); theasinensin A standard (purity > 97%, Nagasaki University, Nagasaki, Japan).

### 4.2. Instruments and Equipment

A 6CR-Z45 tea-rolling machine (Zhejiang Chunjiang Tea Machinery Co., Ltd., Fuyang, China), a 6CHX-70 tea-drying machine (Fujian Anxi Jiayou Machinery Co., Ltd., Quanzhou, China), a BSA124S-CW electronic balance (Sartorius Scientific Instruments Ltd., Göttingen, Germany), an LC-20AD high-performance liquid chromatograph (Shimadzu Corporation, Kyoto, Japan), a 5C18-AR-II column (250 mm × 4.6 mm, 5 μm, Cosmosil Corporation, Kyoto, Japan), a UV-3600 ultraviolet visible near infrared photometer (Shimadzu Corporation, Japan), a 3K15 centrifuge (Shimadzu Corporation, Japan, 4.6 mm, 5 μm, Cosmosil, Japan), a KQ-500DE numerical control ultrasonic cleaner (Kunshan Ultrasonic Instrument Co., Ltd., Shanghai, China), a DHG-9123A electric constant-temperature drying oven (Shanghai Jinghong Experimental Equipments Co. Ltd., Shanghai, China), and a DK-S26 electric constant-temperature water bath (Shanghai Senxin Experimental Instrument Co., Ltd., Shanghai, China) were used.

### 4.3. Experimental Methods

#### 4.3.1. Enzyme Solution Preparation

##### Preparation of Single Enzyme

Fengshui pear enzyme preparation process: pears with the right size and no damage on the skin were selected, then the core was removed and the fruit was cut, pulped, and centrifuged, and finally 10 mL of supernatant was collected as the enzyme solution from Fengshui pear.

Then, 1 g tannase (*Aspergillus niger* source) was weighed and dissolved into 10 mL of water as tannase liquid (*Aspergillus niger* source); 1 g tannase (*Aspergillus oryzae* source) was weighed and dissolved into 10 mL of water as tannase liquid (*Aspergillus oryzae* source); 5 g enzyme laccase was weighed and dissolved into 10 mL of water as laccase liquid. About 10 mL of cellulase liquid and 10 mL of pectinase liquid were directly measured. The commercial tannase was centrifuged at 1500 g for 10 min, and then the supernatant was used as the enzyme solution.

##### Preparation of Combined Enzyme Solution

First, 0.5 g tannase (source of *Aspergillus niger*) was weighed, dissolved into 5 mL of water, and mixed with 5 mL of cellulase solution for the combination of tannase (source of *Aspergillus niger*) and cellulase; 2.5 g of laccase was weighed, dissolved into 5 mL of water, and mixed with 5 mL of cellulase solution for the combination of laccase and cellulase; 0.5 g tannase (source of *Aspergillus niger*) was weighed, dissolved into 5 mL of water, and mixed with 5 mL of pectinase solution; 0.5 g tannase (source of *Aspergillus niger*) was weighed, dissolved into 5 mL of water, and mixed with 5 mL of pectinase solution; 0.5 g of tannase (from *Aspergillus niger*) was weighed, dissolved into 5 mL of water, and mixed with 5 mL of pectinase solution for the combination of both laccase (from *Aspergillus niger*) and pectinase.

#### 4.3.2. Rolled Tea Leaves Preparation

Fresh tea leaves were naturally spread out on a bamboo plaque with a leaf thickness of 5 cm for withering until the moisture decreased to 60~62%. Then they were rolled for 60 min according to the pressurization program of “light pressure, heavy pressure, light pressure” and were immediately shaken off. Finally, rolled tea leaf samples were sealed and stored at −18 °C for analysis.

##### Different Exogenous Enzyme Treatments

First, 300 g of well-decomposed rolled tea leaves was randomly weighed for each of the 11 treatments, and enzyme treatment was carried out by spraying. The enzyme solutions of 10 mL each described in Section 4.3.1 were individually prepared with tannase (*Aspergillus niger* source), tannase (*Aspergillus oryzae* source), fumonisinase, cellulase, pectinase, laccase, cellulase and tannase (*Aspergillus niger* source) combination enzyme, cellulase and laccase combination enzyme, pectinase and tannase (*Aspergillus niger* source) combination enzyme, pectinase and tannase (*Aspergillus oryzae* source) combination enzyme, and pectinase and laccase combination enzyme, and the no enzyme solution was a blank control. After spraying the enzyme solution, the fermentation process was set at the temperature of 32 °C for 4 h, the humidity was controlled at more than 90%, and samples were taken after 2 h and 4 h, respectively, during the fermentation process. After the fermentation process, the drying process was undertaken at a temperature of 120 °C for 15 min for the first drying, then samples were spread for half an hour, and the second drying occurred at 90 °C for 25 min. Finally, processed black tea samples were sealed and stored at −18 °C for analysis.

##### Optimization of Black Tea Fermentation Conditions

First, 300 g of rolled tea leaves was randomly weighed for each portion to carry out the one-way experiment of tea fermentation, the selected experimental factors were: a fermentation temperature of 20 °C, 25 °C, 30 °C, 35 °C, 40 °C, a fermentation time of 1 h, 2 h, 3 h, 4 h, an amount of enzyme of 0.1 g, 0.5 g, 1 g, 2 g, while no enzyme treatment was the control. The humidity was set above 90%. Drying and storage conditions were the same as those in the section “Different Exogenous Enzyme Treatments”. Processed black tea samples were sealed and stored at −18 °C for analysis.

#### 4.3.3. Determination of Moisture Content

National Standard of China GB 5009.3-2016, National Standard for Food Safety, Determination of moisture in food [44] was used to determine the moisture content.

#### 4.3.4. Determination of Tea Polyphenols

National Standard of China GB/T 8313-2018, Determination of total polyphenols and catechins content in tea [45] was used to determine the tea polyphenols.

#### 4.3.5. Detection of Catechins, Theaflavins, Caffeine, and Theasinensin A

Sample pretreatment: “3 Detection of catechins in tea-HPLC method” in National Standard of China GB/T 8313-2018 [45] was used for the determination.

A Shimadzu LC-20AD high-performance liquid chromatograph was used for the analysis. The chromatographic conditions were as follows [46,47]: 5C18-AR-II column (250 mm × 4.6 mm, 5 μm), an injection volume of 10 μL, detection wavelength of 280 nm, flow rate of 0.8 mL/min, column temperature of 35 °C, mobile phase A: 50 mmol/L phosphoric acid, mobile phase B: 100% acetonitrile. Mobile phase A decreased from 96% to 70% at 0~39 min, then decreased from 70% to 25% at 39~54 min, and finally increased from 25% to 96% at 54~55 min. Standard curves of theaflavins were shown in Table S1.

### 4.4. Data Processing and Analysis

One-way analysis of variance (ANOVA), multiple comparisons (Duncan, Kolkata, India, *p* < 0.05), and correlation analysis were performed using SPSS 22.0 software, and GraphPad Prism 9.0 was used for graphing. Experiments were performed in triplicate.

## 5. Conclusions

Black tea is the most consumed tea product in the world, and TF content is an important index related to the quality of black tea samples. If more catechins could be oxidized to form TFs during black tea processing, it might improve the content of TFs and will enhance the quality of black tea.

The process of exogenous tannase could provide an ideal choice to improve the quality of black tea along with regulating theaflavins through the adjustment of the composite of catechin monomers and their decomposition compounds. Exogenous enzymes such as cellulases and proteinases could adjust the dissolution of catechins and other compounds, whilst tannase could change the composition of gallated and non-gallated catechins. After testing tannases, laccase, cellulase, pectinase, and their combinations, tannase from *Aspergillus niger* was selected as the optimal enzyme to increase the content of theaflavins, through promoting the hydrolysis reaction and the benzoylation reaction of catechins, and could result in a theaflavin (TF) content of 1.41%. Our result was consistent with the previous study [35] and could improve the content of TF and significantly enhance the total content of theaflavins during black tea processing, so that it could reduce the bitterness and astringency of tea beverages [34]. The process conditions were optimized with a fermentation time of 3 h, a fermentation temperature of 20 °C, and 1 g of tannase for 300 g of rolled tea leaves.

## Figures and Tables

**Table 1:** Consumption of Catechins under the treatments by Different Exogenous Enzymes during Black Tea Fermentation (%).

Treatments	EGCG		ECG		EGC		EC		Total Catechins	
	Consumption	Decline Rate	Consumption	Decline Rate	Consumption	Decline Rate	Consumption	Decline Rate	Consumption	Decline Rate
E1	4.00 ± 0.07	85.52	2.60 ± 0.03	83.59	0.24 ± 0.02	34.09	0.62 ± 0.03	68.89	7.46 ± 0.03	79.41
E2	3.84 ± 0.08	82.18	2.38 ± 0.04	76.55	0.32 ± 0.01	45.75	0.84 ± 0.02	93.11	7.38 ± 0.03	78.62
E3	3.62 ± 0.09	77.49	2.29 ± 0.03	73.59	0.29 ± 0.03	41.25	0.76 ± 0.01	84.64	6.96 ± 0.04	74.16
E4	3.52 ± 0.02	75.39	2.16 ± 0.02	69.72	0.35 ± 0.01	49.19	0.76 ± 0.04	84.54	6.80 ± 0.02	72.42
E5	3.52 ± 0.02	75.35	2.21 ± 0.02	71.31	0.25 ± 0.02	35.28	0.77 ± 0.02	85.22	6.75 ± 0.02	71.95
E6	3.77 ± 0.01	80.66	2.38 ± 0.02	76.55	0.35 ± 0.01	49.00	0.78 ± 0.01	86.30	7.27 ± 0.03	77.46
E7	3.85 ± 0.03	82.40	2.50 ± 0.04	80.40	0.22 ± 0.01	31.08	0.64 ± 0.03	71.22	7.21 ± 0.03	76.80
E8	3.61 ± 0.07	77.30	2.20 ± 0.02	70.76	0.28 ± 0.01	39.70	0.77 ± 0.02	85.49	6.86 ± 0.03	73.09
E9	3.95 ± 0.04	84.41	2.59 ± 0.04	83.30	0.26 ± 0.01	37.12	0.62 ± 0.02	69.18	7.42 ± 0.03	79.02
E10	3.54 ± 0.07	75.80	2.14 ± 0.03	69.00	0.38 ± 0.02	53.55	0.80 ± 0.03	88.94	6.87 ± 0.02	73.13
E11	3.83 ± 0.03	81.94	2.41 ± 0.01	77.51	0.33 ± 0.01	46.17	0.78 ± 0.02	86.59	7.34 ± 0.02	78.23

Note: E1 is treatment by tannase 1 (tannase from *Aspergillus niger*); E2 is treatment by tannase 2 (tannase from *Aspergillus oryzae*); E3 is treatment by laccase; E4 is treatment by cellulase; E5 is treatment by pectinase; E6 is treatment by enzyme solution from pear; E7 is treatment by both cellulase and tannase 1; E8 is treatment by both cellulase and laccase; E9 is treatment by both pectinase and tannase 1; E10 is treatment by both pectinase and laccase; E11 is the control.

## Data Availability

The datasets generated and/or analyzed during the current study are not publicly available as the data will be used to develop equipment with integrated functions but are available from the corresponding author on reasonable request.

## Supplementary Materials

The following supporting information can be downloaded at: https://www.mdpi.com/article/10.3390/molecules30030452/s1, Table S1: Standard curves of theaflavins. Reference [46] is cited the Supplementary Materials.
