# Supplementary material for: Theaflavins Are Improved by the Oxidation of Catechins in Tannase Treatment During Black Tea Fermentation
Source: Molecules. 2025 Jan 21;30(3):452. doi: 10.3390/molecules30030452 (PMC11820008; doi:10.3390/molecules30030452)
Supplement: Supplementary file 1 [file molecules-30-00452-s001.zip › molecules-3328785-supplementary.pdf]

HPLC analysis of Theaflavins

Table S1 Standard curves of theaflavins

Compound	Linearity range (mg·mL-1)	Recovery %
TF	0.25-5.0	84.14
TF-3-G	0.25-5.0	87.16
TF-3'-G+TFDG	0.50-10.0	105.31

1. Xue, J.J.; Jiang H.Y.; Long D.; Wang, W.W.; Zhang, J.Y. Simultaneous multiresidue determination of theasinensins and theaflavins in tea using high performance liquid chromatography. J. Chinese Inst. Food Sci. Techn. 2014, 14, 5, 237-243.
